# Thought-Controlled Computer Applications: A Brain–Computer Interface System for Severe Disability Support

**DOI:** 10.3390/s24206759

**Published:** 2024-10-21

**Authors:** Kais Belwafi, Fakhreddine Ghaffari

**Affiliations:** 1 Department of Computer Engineering, College of Computing & Informatics, University of Sharjah, Sharjah 26666, United Arab Emirates; 2 Équipes de Traitement de l’Information et Systèmes, UMR 8051, CY Cergy Paris Université, École Nationale Supérieure de l’Electronique et de ses Applications (ENSEA), Centre National de la Recherche Scientifique (CNRS), 95000 Cergy, France; fakhreddine.ghaffari@cyu.fr

**Keywords:** Brain–Computer interface, motor imagery, EEG, brain-controlled operating system, brain-controlled email, brain-controlled internet browser

## Abstract

This study introduces an integrated computational environment that leverages Brain–Computer Interface (BCI) technology to enhance information access for individuals with severe disabilities. Traditional assistive technologies often rely on physical interactions, which can be challenging for this demographic. Our innovation focuses on creating new assistive technologies that use novel Human–Computer interfaces to provide a more intuitive and accessible experience. The proposed system offers four key applications to users controlled by four thoughts: an email client, a web browser, an e-learning tool, and both command-line and graphical user interfaces for managing computer resources. The BCI framework translates ElectroEncephaloGraphy (EEG) signals into commands or events using advanced signal processing and machine learning techniques. These identified commands are then processed by an integrative strategy that triggers the appropriate actions and provides real-time feedback on the screen. Our study shows that our framework achieved an 82% average classification accuracy using four distinct thoughts of 62 subjects and a 95% recognition rate for P300 signals from two users, highlighting its effectiveness in translating brain signals into actionable commands. Unlike most existing prototypes that rely on visual stimulation, our system is controlled by thought, inducing brain activity to manage the system’s Application Programming Interfaces (APIs). It switches to P300 mode for a virtual keyboard and text input. The proposed BCI system significantly improves the ability of individuals with severe disabilities to interact with various applications and manage computer resources. Our approach demonstrates superior performance in terms of classification accuracy and signal recognition compared to existing methods.

## 1. Introduction

Improving access to information for people with disabilities is a priority, as recommended by the United Nations (UN). One way to achieve this is by developing new types of Human–Computer Interface (HCI) [1]. HCI involves designing, implementing, and evaluating interactive systems that meet the user’s needs and tasks [2]. In recent years, a new type of HCI known as the BCI has emerged [3,4,5]. It is designed to assist people with severe disabilities in communicating and interacting with their environment using an alternative interface [6,7].

The rise of BCI technology is transforming how we interact with the world around us. With their portable, convenient, safe, and inexpensive designs, these devices are becoming increasingly accessible to everyday users. Imagine effortlessly controlling your digital environment just by thinking. For example, BCI technology has been proposed to control wheelchair [8,9], smart home control [10], text reader application [11], etc. The BCI establishes a communication channel between the human brain and a computer through mental activity, without requiring physical movement. This mental activity produces changes in electrophysiological signals such as the EEG or Electrocorticogram (ECoG). The BCI system acquires and measures these changes and translates them into control commands.

BCI systems incorporate three primary techniques: vent-Related Desynchronization/Synchronization (ERD/ERS), Steady-State Visual Evoked Potential (SSVEP), and P300. ERD/ERS signifies a decrease or increase in synchronous brain activity in response to an event [12]. P300 is an Event-Related Potential (ERP) response that becomes measurable after 300 ms [13]. It manifests as a positive deflection in voltage when a visual stimulus occurs. SSVEP is an oscillatory wave that responds to visual stimulation at a specific frequency [14].

In order to bridge the significant digital gap between disabled and non-disabled individuals, it is essential to develop innovative assistive technologies using BCIs. These technologies not only promise to enhance accessibility and communication for individuals with disabilities but also aim to empower them with greater independence and integration into the digital world. By focusing on creating and refining BCIs, we can move closer to achieving the ambitious goal of a more inclusive and equitable society where everyone has the opportunity to fully participate in the digital age.

This study proposes a framework to provide a comprehensive computational environment for individuals with severe disabilities and enables users to communicate via email, browse the web, and interact with computer applications such as file systems and process managers. The integrated web browser complies with the Web Accessibility Initiative (WAI) guidelines established by the World Wide Web Consortium (W3C) [15]. This system primarily uses motor imagery in conjunction with visual stimulation to generate brain activity. Users imagine specific limb movements, which produce brain signals that the system detects, filters, classifies, and translates into corresponding actions. Additionally, when text input requires the use of a virtual keyboard, the system seamlessly switches to P300, ensuring a versatile and user-friendly experience. Unlike the system proposed in [16], the suggested finite state machine is controlled through four distinct limb movements. This enhancement not only adds more features but also simplifies the control of the computer and its applications, providing a more intuitive and efficient user experience.

The primary contribution of this study is the proposal of a framework that integrates a computational environment, allowing individuals with severe disabilities to communicate through email, browse the web, and interact with computer applications and operating system components such as file systems and process managers. The study also introduces a sophisticated signal processing chain designed to differentiate between four limb movements using new machine learning algorithms. This four-class classification represents a crucial advancement, as the study rigorously evaluates and compares various machine learning models to identify a chain that not only performs robustly but also significantly outperforms existing approaches in the literature.

The following is the structure of this paper: In Section 2, an overview of related work in various applications controlled by thought is provided. Section 3 presents the methodology, including the proposed framework, feature extraction, and classification techniques. In Section 4, the experimental setup is described, followed by the presentation and discussion of the evaluation results for both the Motor imagery and P300. Finally, Section 5 concludes the paper and discusses potential future research directions.

## 2. Related Work

Several prototypes related to our work utilize EEG signals but do not incorporate the techniques mentioned above or implement a hybrid approach. For example, in [17,18], an application called “Control of an Internet Browser” has been introduced to aid users in navigating web pages. This application allows users to select hyperlinks and scroll through web pages using a matrix of symbols that elicit P300 responses displayed on a separate screen.

Descartes is the first BCI web browser controlled by Slow Cortical Potentials (SCPs) regulation [19]. SCP has two types of shifts, positive and negative. In Descartes, a binary decision tree is constructed for browser commands, where positive SCP shifts are used for selecting and moving the cursor downward, while negative SCP shifts are employed for rejecting and moving the cursor upward. However, Descartes has issues with the selection mechanism, such as the inability to identify the textual label of a picture or icon as a link on a web page.

Neural Signal Surfing Interface (NESSI) is a new EEG-controlled web browser that can be controlled by SCP or SensoriMotor Rhythm (SMR) [20]. NESSI utilizes a binary decision tree similar to Descartes and addresses some of Descartes’ limitations, such as the limited number of web pages, unknown links, graphical links, and identical link names.

In [21], B-explorer is introduced, which allows users to communicate and interact with others via email, SMS messages, and web browsing. The system controls various application actions using eye-blinking signals or P300 responses. Servent et al. developed an EEG BCI web browser controlled by Visual Evoked Potentia (VEP) signals [22]. The screen consists of a browsing frame and a command frame. The BCI web browser can open any web page and interact with its components using a virtual keyboard and a virtual mouse matrix. However, it requires more commands per action than NESSI to handle the web document.

In [16], we presented an integrated computational environment designed to enhance information accessibility and provide assistance to individuals with severe disabilities in their daily tasks. Through the use of customized open-source software tools, this system enables interaction with graphical user interfaces using brain activity, allowing complete control of a personal computer with just two commands. While this approach requires more effort from the user, it maintains a high level of accuracy. The signal processing chain incorporates an Infinite Impulse Response (IIR) filter for artifact removal and utilizes various classifiers, achieving an average accuracy of 94.5% with a Support Vector Machine (SVM) classifier.

Lastly, in [23], BrainBrowser, based on μ rhythm signals over the motor cortex, is proposed. It surpassed Descartes in navigation but could not access all web pages accessible by conventional web browsers as the majority of websites have many components containing links and images, and thus are difficult to present serially due to the amount of information that must be displayed.

## 3. Materials and Methods

**Figure 1:**
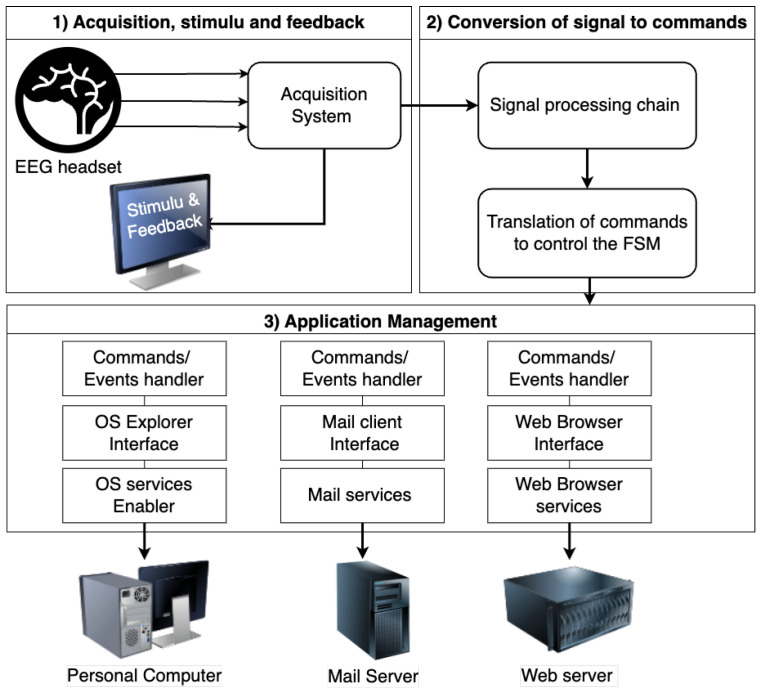
General overview of the proposed framework.

The proposed framework is designed to assist people with severe motor disabilities in communicating via email, browsing the internet, and interacting with their computer’s operating system using their thoughts rather than muscle movements. Figure 1 presents the general overview of the proposed framework, which integrates signal processing algorithms, allowing the detection of the motor imagery signal and the P300 evoked potential to write text, navigate web pages, and develop a command-line interface to manage the Operating System (OS) Explorer.

The system comprises an integrated BCI framework with a translation block for converting the user’s mental activity into control commands via signal processing algorithms such as filtering, feature extraction, and classification methods. The system includes various APIs for controlling its functions, most of which are controlled using motor imagery signals. However, when virtual keyboards are required to enable the user to write texts, the system detects the user’s responses to visual stimuli, which are then analyzed by the BCI framework and translated into artificial actuators. The BCI framework is responsible for acquiring, filtering, recognizing, and classifying these changes and translating them into control commands to manage the integrated system’s applications.

**Figure 2:**
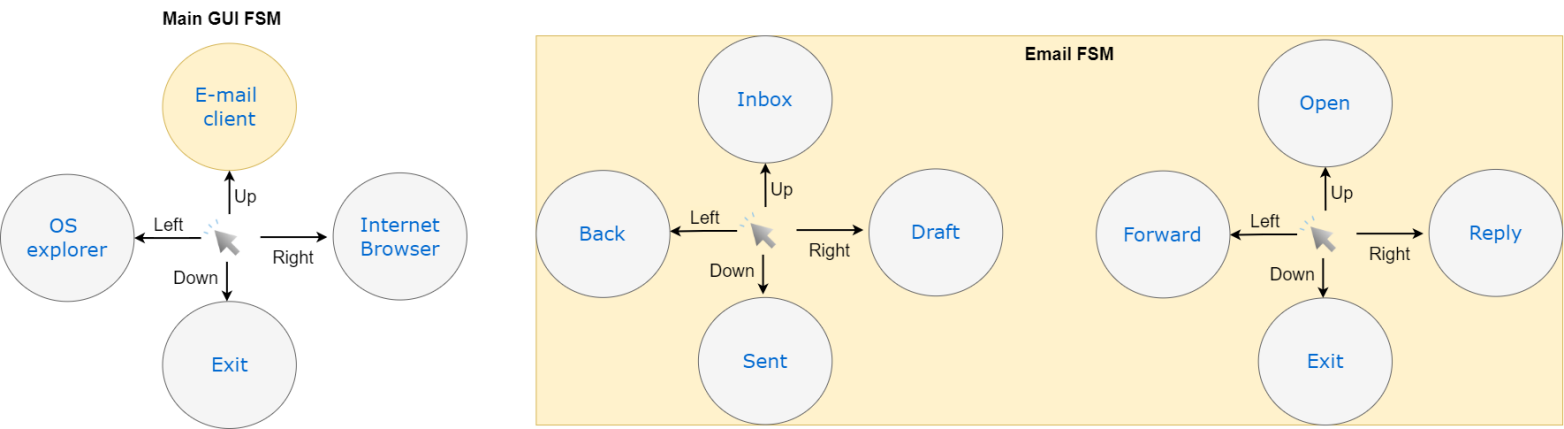
Finite state machine of the proposed framework.

The suggested framework offers diverse and rich services, but the subject only uses four thoughts to control the whole application through a predefined Finite State Machine (FSM) to map these limited control commands into appropriate actions. The proposed FSM is presented in Figure 2, where each user’s choice from an application menu corresponds to a state transition of the FSM. Each imagined limb movement generates a brain activity signal that serves as an input to the FSM to generate a transition from one state to another, along with a possible output.

The subject can use four thoughts, to be mapped into limited control commands, to manage the whole application through a predefined FSM. Figure 2 presents the proposed FSM, where each user’s choice from an application menu corresponds to a state transition of the FSM. Each imagined limb movement will generate a brain activity signal utilized as input to the FSM to generate a transition from one state to another, along with a possible output. Users can use their left hand, right hand, both hands, or rest to interact with the main features of the integrated system. If the user thinks about moving their right hand, the Internet Browser application will be launched. Likewise, if he imagines moving his left hand, the OS explorer will be activated. When the main Graphical User Interface (GUI)-FSM is activated and the user thinks about moving both hands, an email API will launch, allowing the user to control their email account. The application will display the user’s email account folders. The inbox folder will be accessible after the user thinks about moving both hands. The same procedure will be applied to open, reply, forward, and delete emails.

### 3.1. BCI Framework

The BCI framework is responsible for capturing and interpreting brain activity to generate commands or trigger events. EEG signals are usually recorded using an acquisition system that is connected to electrodes placed on a cap covering the user’s scalp. These electrodes are positioned based on the international 10–20 system to cover the brain activities associated with Event-Related Desynchronization/Synchronization (ERD/ERS) and P300. ERD/ERS signifies a decrease or increase in synchronous brain activity when the user thinks about moving one of his limbs [12]. The P300 is an Event-Related Potential (ERP) response that can be observed after 300 ms [13]. This response is seen as a positive change in voltage when a visual stimulus is presented.

The EEG signals are captured, amplified, converted to digital, and then sent to the BCI framework for further signal processing. The proposed framework incorporates signal processing algorithms that enable the detection of motor imagery signals and the P300 evoked potential. This allows for tasks such as writing text, navigating web pages, and developing a command-line interface to manage the OS Explorer. Various EEG signal processing algorithms are chosen and integrated within the BCI framework to enable filtering, feature extraction, and classification of brain activities.

#### 3.1.1. Motor Imagery Processing Chain

**Figure 3:**
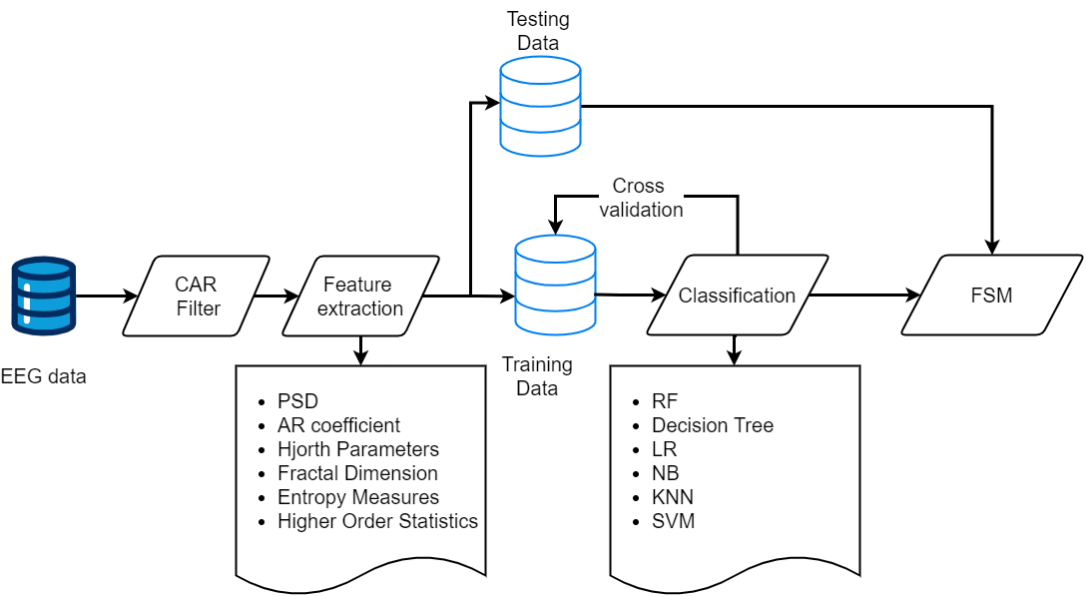
The proposed MI-EEG signal processing chain.

Figure 3 depicts the proposed MI-EEG signal processing chain. Initially, Common Average Reference (CAR) and Laplacian filtering are employed to improve the quality of EEG signals. CAR involves computing the average signal across all electrodes and subtracting it from each electrode’s signal to reduce common noise. For Laplacian filtering, small and large filters are used to enhance signal quality by subtracting the average signal of immediate or further neighboring electrodes. Implementing these methods involved mapping electrode positions, calculating weights, and applying the Laplacian filter to the EEG data for improved neural activity identification.

During the feature extraction stage, we aimed to convert the EEG signals into useful features for our machine learning models. Initially, we aimed to remove time dependency within the features caused by the varying length of trials. We extracted a wide range of features, including the following:

- Power Spectral Density (PSD): Calculated for the μ (8–12 Hz) and β (13–30 Hz) bands to capture frequency-domain information. This is crucial as different mental states and cognitive processes are reflected in distinct frequency bands, aiding in distinguishing between various brain activities. The PSD is computed using the Welch method:
  (1) $$\mathrm{PSD}\left(f\right)=\frac{1}{N}{\left|\sum_{n=0}^{N-1}x\left(n\right)e^{-j2\pi fn/N}\right|}^{2}$$
  where x(n) is the EEG signal in each electrode, *N* is the number of points, and *f* is the frequency.
- Hjorth Parameters: Computed activity, mobility, and complexity for each channel to quantify the signal’s statistical properties:
  (2) $$\begin{array}{cc} \mathrm{Activity} & =\mathrm{Var}(x(t)) \end{array}$$
  (3) $$\begin{array}{cc} \mathrm{Mobility} & =\sqrt{\frac{\mathrm{Var}\left(\frac{dx(t)}{dt}\right)}{\mathrm{Var}(x(t))}} \end{array}$$
  (4) $$\begin{array}{cc} \mathrm{Complexity} & =\frac{\mathrm{Mobility}\left(\frac{dx(t)}{dt}\right)}{\mathrm{Mobility}(x(t))} \end{array}$$
  where x(t) is the EEG signal. These parameters help in understanding the signal’s amplitude variability and frequency content, providing insights into the underlying neural dynamics.
- AutoRegressive (AR) Model Coefficients: Extracted AR model coefficients to capture the temporal structure of the signal, which is useful for modeling its time-dependent properties and identifying consistent patterns over time. The AR model is defined as
  (5) $$x\left(t\right)=\sum_{i=1}^{p}\phi_{i}x\left(t-i\right)+\epsilon \left(t\right)$$
  where $\phi_{i}$ are the AR coefficients, *p* is the order of the model (set to 4), and ϵ(t) is the white noise error term.
- Fractal Dimension and Entropy Measures: Computed fractal dimension, approximate entropy, sample entropy, and permutation entropy to characterize the signal’s complexity:
  (6) $$\begin{array}{cc} \mathrm{Fractal}\;\mathrm{Dimension} & =\lim_{r\to 0}\frac{\log(N(r))}{\log(1/r)} \end{array}$$
  (7) $$\begin{array}{cc} \mathrm{Approximate}\;\mathrm{Entropy} & =\frac{\sum_{i=1}^{N-m}\log\left(C_{i}^{m}\left(r\right)\right)}{N-m+1} \end{array}$$
  (8) $$\begin{array}{cc} \mathrm{Sample}\;\mathrm{Entropy} & =-\log\left(\frac{\sum_{i=1}^{N-m}C_{i}^{m}\left(r\right)}{\sum_{i=1}^{N-m}C_{i}^{m+1}\left(r\right)}\right) \end{array}$$
  (9) $$\begin{array}{cc} \mathrm{Permutation}\;\mathrm{Entropy} & =-\sum p(\pi )\log(p(\pi )) \end{array}$$
  where N(r) is the number of data points within radius *r*, $C_{i}^{m}\left(r\right)$ is the correlation integral, and p(π) is the probability of permutation pattern π. These measures are valuable for assessing the irregularity and unpredictability of the EEG signals, which are indicative of different cognitive states.
- Higher Order Statistics: Calculated skewness, kurtosis, variance, and standard deviation for each channel:
  (10) $$\begin{array}{cc} \mathrm{Skewness} & =\frac{1}{N}\sum_{i=1}^{N}{\left(\frac{x_{i}-\bar{x}}{\sigma }\right)}^{3} \end{array}$$
  (11) $$\begin{array}{cc} \mathrm{Kurtosis} & =\frac{1}{N}\sum_{i=1}^{N}{\left(\frac{x_{i}-\bar{x}}{\sigma }\right)}^{4}-3 \end{array}$$
  (12) $$\begin{array}{cc} \mathrm{Variance} & =\frac{1}{N-1}\sum_{i=1}^{N}{\left(x_{i}-\bar{x}\right)}^{2} \end{array}$$
  (13) $$\begin{array}{cc} \mathrm{Standard}\;\mathrm{Deviation} & =\sqrt{\mathrm{Variance}} \end{array}$$
  where $x_{i}$ are the data points, $\bar{x}$ is the mean, σ is the standard deviation, and *N* is the number of data points. These statistics provide detailed information about the distribution and variability of the EEG signals, which can be used to identify abnormalities and subtle differences in brain activity.

By compiling and normalizing these features, we created a robust feature set for training our machine learning models. This approach significantly improved the accuracy and reliability of our models, leading to better performance in classifying EEG signals.

In the classification stage, we evaluated multiple machine learning models with our dataset. We split the data into training and testing sets to ensure robust model evaluation. The training set, comprising 80% of the data, was used to fit the models, while the remaining 20% was reserved for evaluating their performance. This allowed us to assess how well each model generalized to unseen data. The classifiers tested included Random Forest (RF), K-Nearest Neighbors (KNN), SVM, Naive Bayes (NB), Logistic Regression (LR), and Decision Tree (DT), chosen for their distinct advantages in handling EEG signal classification complexities.

- RF: This ensemble method combines multiple decision trees to improve prediction accuracy and robustness against overfitting. It is well suited for capturing the non-linear relationships within EEG data.
- KNN: A simple, yet effective, non-parametric method that classifies data based on the majority vote of its neighbors. It excels in capturing the local structure of the EEG features.
- SVM: Known for its effectiveness in high-dimensional spaces, SVM constructs hyperplanes to separate different classes with maximal margin, enhancing the model’s ability to generalize from the training data. The One-Versus-Rest (OVR) approach, in combination with majority voting, is applied because this algorithm discriminates between only two classes.
- NB: This probabilistic classifier assumes independence between features and applies Bayes’ theorem, offering a fast and efficient approach for real-time classification of EEG signals.
- LR: A straightforward and interpretable method used to model the probability of a binary response based on one or more predictor variables. It is useful for its simplicity and quick computation.
- DT: This model makes decisions by splitting the data into subsets based on the value of input features. It is intuitive and easy to interpret, making it valuable for understanding the decision-making process.

By testing these diverse models, we aimed to identify the optimal classifier that balances accuracy, computational efficiency, and the ability to generalize across different users and conditions.

#### 3.1.2. P300 Processing Chain

The subject interacts with the integrated systems using the keyboard to input commands. An efficient alternative to typing is the P300 speller, which relies on gaze movements alone. The signal processing chain of the P300 speller is not discussed in this paper, but further details can be found in [16]. We are focusing on a specific part of the EEG signals that occur after each intensification, aiming to create “high-level” features for our classifier. To do this, we first collected data from each channel, looking at the samples taken from 0 to 667 ms after the intensification started. We know that evoked potentials show up about 300 ms after the stimulus, so this time window should be ample to capture the necessary features for effective classification. Next, we filtered each extracted signal using an 8th-order Chebyshev Type I bandpass filter, with cutoff frequencies set at 0.1 and 10 Hz, and then we decimated the data based on the higher cutoff. After these steps, each signal from a single channel consists of 14 samples. These 14 samples from all 64 channels were combined into a single vector for each subject. The training set for each subject consists of 15,300 post-stimulus vectors, each with a dimension of 896, and labeled as either 0 or 1. The study addresses a complex classification problem in two phases. The first phase involves a two-class classification to predict if a signal corresponds to a P300 response. The second phase deals with a multi-class classification problem where the aim is to predict a symbol/command from a command matrix.

### 3.2. Integrated System

The following component is tasked with receiving commands and events from the BCI framework and executing the appropriate actions while providing feedback on the screen. The same command or event may lead to different actions depending on the specific context of the application. The application manager ensures that only the active application handles the received event. The command/event handler carries out the necessary tasks at the application level in response to the user’s requests. The application interface displays the various services that users can access along with the associated feedback. The overall environment encompasses three applications.

#### 3.2.1. An E-Mail Client Application:

A prototype for an email client app was created using the BCI2000 framework. This prototype utilizes brain activity signals as visual cues for command execution. However, accuracy issues were encountered in well-lit environments and on small screens. Additionally, the speed of message writing was found to be very slow, averaging three to four characters per minute. To address these issues, the app was redesigned using the ERD/ERS technique, and several improvements were implemented:

- Full Email Folder Access: Users can now easily access all email account folders, including Inbox, Sent, Spam, Trash, Drafts, and Contacts.
- Improved Message Writing Speed: The addition of an auto-complete feature has significantly sped up message composition.
- Ready-Made Templates: The app includes pre-written message templates for common occasions like birthdays and holidays, making it more convenient.

#### 3.2.2. An Internet Browser

The proposed internet browser complies with the guidelines specified in the Web Accessibility Initiative [15] and fully satisfies the requirements for enabling “true web access”, as indicated in [24]. These requirements include the following:

- Ensuring that selected links are visible.
- Allowing traversal of the history list both forward and backward.
- Providing full user access for updating bookmarks.
- Offering alternatives for filling out forms.
- Providing helpful information about link targets to the user.

More details about the proposed internet browser interface can be found in [25].

#### 3.2.3. An OS Explorer

This application offers a user-friendly interface for seamless interaction with computers. Users can input command-line statements to efficiently manage various aspects of the operating system, such as the file system, processes, and system configurations. To enhance user experience and usability, a GUI has been integrated, with its design detailed in [16]. Our objective is to provide access to software programs, including the MS Office suite, installed on the user’s computer. Additionally, the application incorporates an innovative feature that allows users to control the computer mouse or initiate keyboard inputs using EEG signals, enabling the launching of specific applications.

## 4. Results and Discussion

The proposed framework allows users to manage their computer resources by controlling an email client, web browser, e-learning tool, and both command-line and graphical user interfaces. It offers essential functionalities for each application. Control is achieved through brain activity, using an EEG signal processing chain verified with a public dataset for motor imagery and P300. Classification is carried out using new machine learning algorithms and classic machine learning algorithms such as RF, DT, LR, NB, KNN and SVM. Accuracy metrics are used to measure correctly classified trials during the testing phase.

### 4.1. Description of the Dataset

The proposed approach was validated using two distinct public datasets labeled by professional experts: one for motor imagery and one for P300 signals. These datasets provided comprehensive data for evaluating the effectiveness of our framework.

#### 4.1.1. Motor Imagery Dataset

The MI-EEG data used in this study is from a dataset provided in [26], with 62 healthy subjects performing motor imagery tasks: left hand, right hand, both hands and rest. This dataset includes EEG recordings from 62 EEG channels placed on the skull according to the international 10–10 system. Subjects underwent 7–11 BCI training sessions to control a computer cursor in 1D (left/right, up/down) and 2D spaces using their “intent”. The data were collected over several sessions, providing a rich longitudinal dataset with over 600 h of EEG recordings. The dataset is notable for its size and the number of trials, making it highly valuable for training and evaluating machine learning models for the proposed BCI applications.

#### 4.1.2. P300 Dataset

The P300 dataset is recorded using the P300 speller based on the oddball paradigm. It states that rare, expected stimuli produce a positive deflection in the EEG after about 300 ms [27]. In order to spell a single character, each of the 12 rows and columns of the matrix is randomly intensified into a sequence. The participant is instructed to concentrate on the character they wish to spell, and then an evoked potential (document number 1) appears in the EEG in response to the intensification of a row or column containing the desired character. To make the spelling procedure more reliable, this sequence of intensifications is repeated 15 times for each character to spell. Two healthy subjects participated in this experiment in five different sessions, each composed of different runs where each run corresponds to spelling a word. The EEG signals were acquired using 64 channels placed according to the international localization 10–20 system. Each subject’s training set has 85 characters, with 180 post-stimulus labeled signals collected using 64 channels for each character [5,16,28].

Both EEG datasets were cleaned to remove noise and artifacts, normalize the signals, segment the data, and extract relevant features. Steps like these enhance the accuracy and reliability of the results. Additionally, missing data were handled by discarding incomplete records and samples without necessary EEG electrodes. Finally, the data from multiple sources were combined into a single dataset by standardizing the annotation formats, providing a solid foundation for subsequent data processing.

### 4.2. Results of the Experiments

We created multiple models to classify EEG signals into four classes: RIGHT, LEFT, UP, and DOWN. Table 1 summarizes the obtained performances for the different algorithms. Firstly, the RF model with 100 trees is used, and it performed well, achieving 70% accuracy, as shown in Table 1. Then, KNN model with k=5 is implemented, which leverages similarities between instances to classify motor imagery tasks accurately. The obtained average accuracy reached about 62%.

Additionally, a Gaussian NB model was developed, utilizing probabilistic reasoning for classification. The results are listed in Table 1. A SVM with a linear kernel was also employed, and cross-validation was performed to find the optimal hyperplane for separating the classes. The performance of this model is approximately 79%.

Moreover, LR with L2 regularization was used to effectively model the probability of the classes, with the highest accuracy of about 82%. Lastly, a DT model with a maximum depth of 10 was developed to capture the decision rules directly from the extracted features, resulting in the performance metrics shown in Table 1.

**Figure 4:**
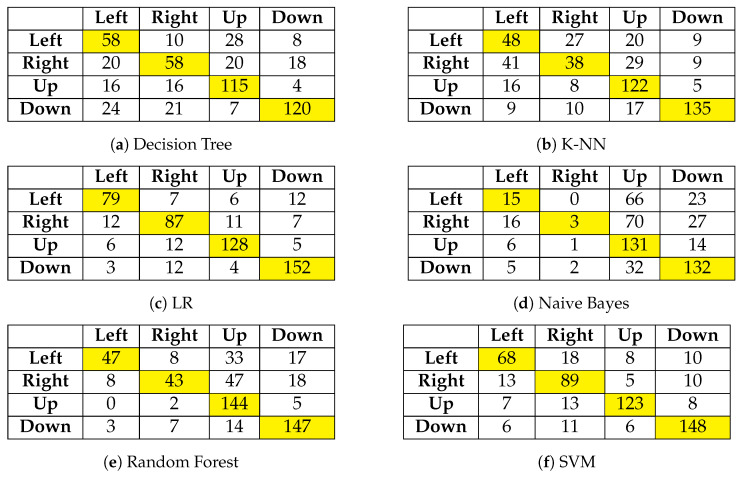
Confusion Matrices for Different Models.

In the realm of classifying four-class motor imagery, LR demonstrated the highest accuracy. However, SVM and RF models also proved to be strong contenders, exhibiting high precision and recall with fewer misclassifications. In contrast, the DT, KNN, and NB models encountered more challenges, particularly in distinguishing between Class 1 (RIGHT) and Class 2 (LEFT), as shown in Figure 4. While LR emerged as the top choice for accuracy, SVM and RF are also excellent options due to their strong performance across all metrics.

The experiments demonstrated that the ensemble of RF classifiers achieved the highest accuracy compared to the other classifiers. The proposed approach attained an average accuracy rate of 95% using the RF classifier, while using the same signal processing chain and the NB classifier resulted in an average accuracy of 85%. These results outperformed those obtained by the second and third-place winners of the BCI competition, as shown in Table 2.

The strong accuracies indicated the effectiveness of the proposed approach, as the accuracy exceeded the navigation accuracy requirement by 20% [33]. Therefore, the experimental results supported the use of the provided framework by individuals with severe disabilities, as it guaranteed good performance in accuracy.

A comparison of the proposed work with relevant studies is provided in Table 2. The comparison is conducted using the same dataset for benchmarking purposes. This Table includes the methods employed and the accuracies obtained, showcasing how the suggested approach stands in relation to established methodologies.

For the Motor Imagery task, our approach using CAR and Statistical features combined with a RF classifier achieved an accuracy of 82.10%. This outperforms the method utilized by [29], which employed Laplacian, Common Spatial Pattern (CSP), and Linear Discriminant Analysis (LDA), resulting in an accuracy of 63.37%. Similarly, our method surpasses the approach proposed in [31], which utilized deep classification models like EEGNet, Shallow & Deep ConvNet, MB3D, and ParaAtt, achieving 74%. However, the method by [30], which applied Spatial filter, PSD, and Canonical Variate Analysis (CVA) along with Decoder Calibration, obtained a competitive accuracy of 80%, slightly lower than our method.

For the P300 task, our proposed method using IIR filtering, Decimation, and an RF classifier achieved the highest accuracy of 95%. This is slightly higher than the results presented in [27], which used FIR filtering, Decimation, and SVM, achieving an accuracy of 94.75%. Additionally, our method also outperforms the approach of [16], which employed IIR filtering, Decimation, and SVM, with an accuracy of 94.50%. The method utilized by [32], which included IIR filtering, Channel selection, and SVM, resulted in a notably lower accuracy of 80%.

These comparisons highlight the effectiveness of our methods in both Motor Imagery and P300 tasks, demonstrating improvements over several established techniques in the literature. By employing advanced preprocessing and classification strategies, our approach achieves superior performance, providing a robust solution for EEG signal classification. The accuracy of these methods can be improved by selecting the appropriate filter parameters for artifact removal [34]. Inadequate tuning of the filter parameters can negatively impact accuracy. Additionally, integrating the CAR filter can enhance the signal quality in each channel. Finally, optimizing classifier parameters, such as the number of hidden layers, is also likely to lead to better outcomes.

Introducing four options offers users greater flexibility when interacting with the operating system. While a two-class classification system is straightforward to implement and can improve accuracy in distinguishing between two actions, it comes with limitations, such as a restricted number of available actions. For instance, using only two classes requires users to follow a specific finite state machine to open their email, which involves at least two motor imagery actions. In contrast, our proposed system allows users to open their email with just one movement, creating a more fluid interface and greater flexibility. This approach not only reduces cognitive load but also minimizes fatigue, both of which are crucial considerations.

Numerous challenges were encountered during this study, significantly impacting the development and implementation process. One major difficulty was ensuring data quality and availability. The lack of annotated datasets posed a significant obstacle. Integrating data from various sources required standardizing inconsistent formats, adding complexity to the data preparation process. Furthermore, the EEG signals frequently contained noise from physiological artifacts, such as eye blinks and muscle movements, as well as external electrical interference. Addressing these issues necessitated the use of advanced filtering and preprocessing techniques such as the ICA [35], PCA [36], WOLA [37], adaptative filtering [38], wavelet [39], etc.

Training and validating our machine learning models proved to be computationally intensive, requiring significant resources. Ensuring that our models generalized well to different subjects and conditions was also challenging, requiring extensive testing and validation. Despite these challenges, the study made significant progress in improving EEG signal processing and machine learning model performance. The insights gained from addressing these difficulties will guide future work and contribute to the advancement of EEG-based systems.

The proposed framework demonstrates versatility beyond its primary application in controlling computer applications like Email, Web Browser and OS explorer. It can be effectively adapted for functional substitution in various domains such as the control of a wheelchair merely by thinking, providing robust solutions where precision and reliability are crucial. Moreover, the framework’s potential extends to pathology analysis, where its ability to process and interpret complex data can aid in diagnosing and understanding diseases. The BCI technology can be harnessed for in-depth analysis of various pathologies such as autism spectrum disorder, predictive capabilities for epileptic seizures, Alzheimer’s disease, and sleep disorders. Irrespective of the application, the signal processing chain remains consistent as it is composed of signal acquisition, EEG filtering, feature extraction and classification. By leveraging its adaptable architecture, the framework can be customized to meet the specific needs of different fields, making it a valuable tool for a wide range of applications, from healthcare to advanced scientific research.

Usually, the use of BCI causes fatigue and increases the cognitive load, especially for users with severe disabilities. While these technologies offer great promise for improving communication and control, it is important to understand that the mental effort needed to operate them can be significant, especially during the use of a P300 speller requiring gaze focusing. Evaluating BCIs should include a thorough assessment of user comfort and cognitive demands, ensuring that the benefits do not lead to increased stress or fatigue. Developing user-friendly interfaces and providing adequate support can help address these challenges, making the technology more effective and sustainable for all users.

## 5. Conclusions

This research introduces a comprehensive BCI framework created to help people with severe disabilities interact with computer APIs merely by thinking. The framework integrates two signal processing chains to detect and process P300 and motor imagery signals. It includes CAR and Laplacian filters for signal enhancement. Feature extraction involves power spectral density, Hjorth parameters, autoregressive model coefficients, fractal dimension, entropy measures, and higher-order statistics, ensuring reliable results with cross-validation. It also uses various classification models such as Random Forest, K-Nearest Neighbors, Support Vector Machine, Naive Bayes, Logistic Regression, and Decision Tree. Logistic Regression showed the best accuracy, with SVM and Random Forest also performing well. Despite more misclassifications with Decision Tree, K-NN, and Naive Bayes, the system effectively classified EEG signals, paving the way for future enhancements and real-world applications. The system achieved an average classification accuracy of approximately 82% for motor imagery signals and a 95% recognition rate for P300 signals for two users, demonstrating its effectiveness in translating EEG signals into actionable commands.

The focus of future work will be on reducing the error rate in the EEG signal processing chain to enhance system reliability and validate the system according to the online approach to study the effect of the feedback on users’ brain activity. Furthermore, we will introduce new features to the proposed APIs to improve user experience, allowing individuals to gain full control of the computer simply by using their thoughts.

## Figures and Tables

**Table 1:** Performance metrics for different classification algorithms.

Algorithm	Accuracy	Precision	Recall	F1 Score
Motor imagery				
RF	0.701	0.736	0.666	0.666
DT	0.646	0.630	0.630	0.630
LR	0.821	0.810	0.810	0.810
NB	0.5174	0.490	0.450	0.390
KNN	0.6206	0.590	0.590	0.590
SVM	0.7882	0.780	0.770	0.770
P300				
RF	0.950	0.94	0.93	0.94
DT	0.89	0.88	0.87	0.88
LR	0.91	0.90	0.89	0.90
NB	0.85	0.84	0.83	0.84
KNN	0.880	0.87	0.86	0.87
SVM	0.945	0.949	0.941	0.945

**Table 2:** Comparison of Our Work with Literature.

Dataset	Reference	Method	Accuracy (%)
Motor Imagery	[29]	Laplacian, CSP, LDA	63.37
	[30]	Spatial filter, PSD, CVA, Decoder Calibration	80.00
	[31]	MI-EEG deep classification models: EEGNet, Shallow & Deep ConvNet, MB3D and ParaAtt	74.00
	Proposed method	CAR, Statistical features, RF	82.10
P300	[16]	IIR, Decimation, SVM	94.50
	[27]	FIR filter, Decimation, SVM	94.75
	[32]	IIR, Channel selection, SVM	80.00
	Proposed method	IIR, Decimation, RF	95.00

## Data Availability

Data are contained within the article.

## Abbreviations

The following abbreviations are used in this manuscript:

API	Application Programming Interface
AR	Autoregressive
BCI	Brain–Computer Interface
CAR	Common Average Reference
DT	Decision Tree
EEG	Electroencephalogram
ECoG	Electrocorticogram
ERD/ERS	Event-Related Desynchronization/Synchronization
ERP	Event-Related Potential
FSM	Finite State Machine
GUI	Graphical User Interface
HCI	Human–Computer Interfaces
IIR	Infinite Impulse Response
KNN	K-Nearest Neighbors
LR	Logistic Regression
MI	Motor Imagery
NB	Naive Bayes
NESSI	Neural Signal Surfing Interface
OS	Operating System
PSD	Power Spectral Density
RF	Random Forest
SCP	Slow Cortical Potentials
SMR	SensoriMotor Rhythms
SSVEP	Steady-State Visual Evoked Potential
SVM	Support Vector Machine
UN	United Nations
VEP	Visual Evoked Potential
W3C	World Wide Web Consortium
